# HMQ‐T‐B10 induces human liver cell apoptosis by competitively targeting EphrinB2 and regulating its pathway

**DOI:** 10.1111/jcmm.13729

**Published:** 2018-09-14

**Authors:** Bingling Dai, Xianpeng Shi, Nan Ma, Weina Ma, Yanmin Zhang, Tianfeng Yang, Jie Zhang, Langchong He

**Affiliations:** ^1^ School of Pharmacy Health Science Center Xi'an Jiaotong University Xi'an China

**Keywords:** apoptosis, EphrinB2, HMQ‐T‐B10, liver cancer cell

## Abstract

Hepatocellular carcinoma (HCC) is a highly prevalent cancer worldwide and it is necessary to discover and develop novel preventive strategies and therapeutic approaches for HCC. Herein, we report that EphrinB2 expression is correlated with liver cancer progression. Moreover, by using phosphorylated proteomics array, we reveal a pro‐apoptosis protein whose phosphorylation and activation levels are up‐regulated upon EphrinB2 knockdown. These results suggest that EphrinB2 may act as an anti‐apoptotic protein in liver cancer cells. We also explored the therapeutic potential of HMQ‐T‐B10 (B10), which was designed and synthesized in our laboratory, for HCC and its underlying mechanisms in vitro and in vivo. Our data demonstrate that B10 could bind EphrinB2 and show inhibitory activity on human liver cancer cells. Moreover, induction of human liver cancer cell apoptosis by B10 could be augmented upon EphrinB2 knockdown. B10 inhibited HCC cell growth and induced HCC cell apoptosis by repressing the EphrinB2 and VEGFR2 signalling pathway. Growth of xenograft tumours derived from Hep3B in nude mice was also significantly inhibited by B10. Collectively, these findings highlight the potential molecular mechanisms of B10 and its potential as an effective antitumour agent for HCC.

## 1 INTRODUCTION

EphrinB2 is a cell surface transmembrane protein encoded by the EFNB2 gene in humans.^1^ It is widely expressed in tumour cells and mediates tumour cell proliferation, invasion and migration.^2^, ^3^ EphrinB2 can activate several Eph receptors (termed “forward” signalling), but can also serve as a receptor (“reverse signalling”). The reverse signal activity promotes tumourigenesis and epithelial‐mesenchymal transition through its signalling molecules for tyrosine phosphorylation sites and a PDZ binding motif in the EphrinB2 cytoplasmic domain.^4^ EphrinB2 has been shown to undergo internalization and is involved in VEGFR2‐ and VEGFR3‐mediated angiogenesis in cultured cells. EphrinB2 promotes VEGFR endocytosis in endothelial cells and enhances VEGF‐mediated angiogenesis.^5^, ^6^ Regulation of VEGFR signalling in cancer cells further results in the activation of PI3K/AKT and MAPK/ERK pathways which regulate cell proliferation, migration, and angiogenesis. EphrinB2 has been verified as a poor prognostic indicator in several solid tumours including pancreatic adenocarcinoma, bladder urothelial carcinoma, and thyroid carcinoma.^7^, ^8^, ^9^

Hepatocellular carcinoma (HCC) is a major type of primary liver cancer with an annual incidence of more than half a million new cases worldwide,^10^, ^11^ ranking as the fifth most frequently diagnosed malignant tumour and the third leading cause of cancer‐related death globally.^12^ Attributed to its dismal prognosis, high mortality, and rapid progression and metastasis, liver cancer is still associated with severe disease‐ and treatment‐related morbidity.^13^ New drugs are being developed that work differently from standard chemotherapeutic drugs. These targeted drugs act on specific parts of cancer cells or their surrounding environment. Sorafenib, which acts as a multiple tyrosine kinase inhibitor, is a mainstream molecular targeted drug approved for HCC treatment.^14^, ^15^, ^16^ Previous studies have identified multiple mechanisms underlying reduced sensitivity to sorafenib in HCC,^17^ including various molecular and signalling pathway alterations such as activation of the EGFR pathway,^18^ epithelial mesenchymal transition^19^ and induction of autophagy.^20^, ^21^ Demonstrations of the efficacy of targeted molecular therapies have triggered the search for additional molecules with therapeutic potential in HCC.

**Figure 1:**
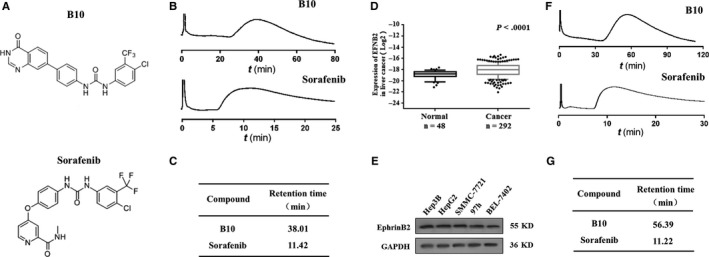
EphrinB2 is correlated with liver cancer and B10 suppresses liver cancer cell proliferation and colony formation. A, Chemical structure of B10 and sorafenib. B, CMC chromatograms of B10 and sorafenib on a VEGFR2/HEK293 CMC column. C, The retention time of B10 and sorafenib on CMC. D, Expression of EFNB2 gene in the TCGA database between normal tissues and clinical liver tissues. **P <* *.001 vs the normal group. E, Expression of EphrinB2 in liver cancer cells including Hep3B, SMMC‐7721, HepG2, Bel‐7402 and 97 h. F, CMC chromatograms of B10 and sorafenib on an EphrinB2/HEK293 CMC column. G, The retention time of B10 and sorafenib on CMC

In this study, we explored EphrinB2 as a promising marker for HCC prognosis and therapy. Its association with HCC clinical characteristics and the potential underlying mechanisms were explored. Moreover, we aimed to reveal the functions and mechanisms of B10 (Figure 1A), which was synthesized in our laboratory, in modulating the in vitro and in vivo growth of HCC. We found that B10 inhibited HCC cell proliferation, induced HCC cell apoptosis, and suppressed xenograft growth in nude mice by targeting EphrinB2 and its pathway.

## 2 MATERIALS AND METHODS

### 2.1 Reagents

Sorafenib was ordered from Dalian Meilun Biotech Co., Ltd (Dalian, China) and a novel agent, B10 (purity >98%), was designed and synthesized by Jie Zhang's group in the Research and Engineering Center for Natural Medicine Xi'an Jiaotong University.^22^, ^23^ All compounds were dissolved in DMSO for use in biochemical assays. DMEM, RPMI‐1640, F12 medium, MTT, DMSO and Hoechst 33258 were obtained from Sigma‐Aldrich (St. Louis, Missouri, USA), Foetal Bovine Serum (FBS) from HyClone (Logan, Utah, USA) and Trypsin from Amresco (Solon, Ohio, USA). Penicillin was purchased from General Pharmaceutical Factory (Haerbin, China) and streptomycin from North China Pharmaceutical (Shijiazhuang, China). G418 was obtained from Gibco (Carlsbad, CA, USA), VEGFR2 kinase from Carna Biosciences (Kobe, Japan), HTRF VEGFR2 kinase kit from Cisbio (Codolet, France) and Annexin V‐FITC reagent kit from KeyGEN BioTECH Corp., Ltd (Jiangsu, China). p44/42 MAPK (ERK1/2), phospho‐p44/42 MAPK(p‐ERK1/2), VEGFR2, phosphor‐VEGFR2 and phospho‐mTOR rabbit monoclonal antibodies (mAbs) were purchased from Cell Signaling (Boston, MA, USA). VEGFR3, mTOR, Bax, and Bcl‐2 rabbit polyclonal antibodies (polyAbs) were obtained from Protein Technology Group (Chicago, IL, USA). AKT, phosphor‐AKT, and EphrinB2 rabbit mAbs were from Epitomics (Burlingame, CA, USA). Rabbit anti‐GAPDH, goat anti‐rabbit IgG, BCA protein assay reagent kit, and enhanced chemiluminescent (ECL) plus reagent kit were obtained from Pierce Biotechnology (Rockford, IL, USA). Protease inhibitor cocktail and phosphatase inhibitor cocktail were purchased from Roche Technology (Basle, Switzerland, USA). The TRIzol reagent and Lipofectamine 2000 reagent were purchased from Invitrogen (Carlsbad, CA, USA). Prime Script RT Master Mix Perfect Real Time kit and SYBR^®^ Premix Ex Taq TM II were purchased from Takara biotechnology (Dalian, China). The RNA oligo was purchased from Gene Pharma (Shanghai, China). Other reagents used were of analytical grades.

### 2.2 Cell culture and animals

Human SMMC‐7721, Hep3B, HepG2, Bel‐7402 and 97 h liver cancer cell lines were purchased from Shanghai Institute of Cell Biology in the Chinese Academy of Sciences (Shanghai, China). HEK293 cells were obtained from Professor Xu Li. EphrinB2/HEK293 and VEGFR2/HEK293 cell lines which over‐expressed EphrinB2 and VEGFR2 were constructed at the Research and Engineering Center for Natural Medicine, Xi'an Jiaotong University. Hep3B, HepG2, and HEK293 cell lines were cultured in DMEM medium with 10% (v/v) FBS and SMMC‐7721, Bel‐7402 and 97 h cell lines were cultured in RPMI‐1640 medium with 10% (v/v) FBS. EphrinB2/HEK293 and VEGFR2/HEK293 cells were maintained in DMEM medium supplemented with 10% FBS and 200 mg/mL G418. All cell lines were incubated in a humidified atmosphere containing 5% CO_2_ at 37°C.

Four‐to‐six‐weeks‐old BALB/C nude male mice were purchased from Shanghai Super‐BK laboratory animal Co., Ltd. (Shanghai, China) and housed in the Experimental Animal Center of Xi'an Jiaotong University. The entire procedure was carried out in accordance with the approved guidelines of the regional authorities, according to China animal‐care regulations. Animal care was in accordance with the National Institute of Heath guidelines and the Animal Research Committee of Xi'an Jiaotong University (SYXK shaan 2015‐002). All animal experiments were carried out according to the guidelines and approval of the Institutional Animal Care and Use Committee of Xi'an Jiaotong University.

### 2.3 Preparation of CMSP (cell membrane stationary phase)

VEGFR2/HEK293 and EphrinB2/HEK293 cells were harvested and re‐suspended in 50 mmol/L Tris‐HCl (pH 7.4), followed by ultrasonic destruction for 30 minutes. The homogenate was centrifuged and the precipitate was then suspended in 5 mmol/L PBS. The CMSP was prepared by adsorption of the cell membrane suspension (5 mL) on the activated silica (0.05 g) under vacuum and with a gentle agitation. Finally, the mixture obtained was packed into a column (10 × 2.0 mm I.D.) using a wet packing method (10 MPa, 5 minutes). The entire procedure was performed at 4°C. CMC (cell membrane chromatography) analysis was performed on a Shimadzu. LC‐20A apparatus that consisted of two LC‐20AD pumps, a DGU‐20A3 degasser, a SIL‐20A auto sampler, a CTO‐20A column oven, and an SPD‐M20A diode array detector (Shimadzu, Kyoto, Japan). The data were acquired using the LC solution software (Shimadzu, Kyoto, Japan). The detection wavelength used was 270 nm. The chromatographic conditions were as follows: CMC column 10.0 mm × 2.0 mm; flow rate 0.6 mL/min; column temperature 37°C; mobile phase 50 mmol/L phosphate‐burred saline, pH 7.4.

### 2.4 Lance assay for VEGFR2 kinase activity

VEGFR2 kinase was determined using LANCE^®^ *ultra* TR‐FRET kinase assay protocol (PerkinElmer Life and Analytical Sciences, Shelton, CT, USA). In total, 2 μL of each, VEGFR2 kinase and substrate, was added to the 384‐well plate, and 4 μL B10 at various concentrations was then added to the assay plate. Subsequently, 2 μL ATP was added and the reaction was allowed to proceed at 37°C for 30 minutes (the optimized concentrations of reaction system were as follows: 0.003767 ng/μL VEGFR2 kinase, 1.332 μmol/L ATP and 121.4 nmol/L substrate). The TK‐antibody labelled with Eu^3+^‐cryptate and streptavidin‐XL665 was added with EDTA (used to stop the kinase activity) to detect the phosphorylated product after incubation at room temperature for 1 hour. Further, the fluorescence of the resulting solution was measured at 665 and 615 nm using the multilabel plate reader of Perkin‐Elmer victor 5. The kinase activity was expressed by the ratio of A665 × 10^4^/A615. IC50 values were calculated by Prism software.

### 2.5 Cell viability assay

SMMC‐7721, Hep3B, Bel‐7402, HepG2 and 97 hours cells were seeded into 96‐well plates and various concentrations of B10 and sorafenib were added; the plates were incubated for 48 hours. MTT solution (5 mg/mL) was added and the plates were incubated for another 4 hours, followed by measurement at 490 nm on a microplate reader (Bio‐Rad Instruments, Hercules, CA, USA). Results were expressed as the percentage of cell viability ratio. Percentage of cell viability ratio = [1−(OD_treatment group_ − OD_blank group_)/(OD_control group_ − OD_blank group_)] × 100%.

### 2.6 Colony formation assay

Hep3B, SMMC‐7721, HepG2, Bel‐7402, and 97 hours cells were seeded in 12‐well plates (200 cells per well) overnight, followed by addition of fresh medium with or without B10 and incubation of the plate for 48 hours. Then the plates were cultured for an additional 10‐15 days until the colonies were clearly visible and countable. Colonies were stained with crystal violet for visualization and counting. After washing the plates, images of the plates were obtained through the chemiluminescent and fluorescent imaging system (Champchemi Professional, SG2010084, Sage Creation, Beijing, China).

### 2.7 Phospho‐antibody microarray analysis

The expression profile of 12 signalling pathway phosphor‐related proteins was detected and analysed using a human CSP100 Antibody Array kit (Full Moon Biosystems, Sunnyvale, CA, USA). Protein microarray analysis was carried out as per the manufacturer's instructions (Wayen Biotechnologies, Shanghai, China). Briefly, cell lysates obtained from Hep3B cells, B10‐treated Hep3B cells, and EphrinB2 siRNA Hep3B cells were added to the array. The array contains 269 antibodies, each of which has 6 replicates that are printed on standard‐size coated glass microscope slides. Briefly, the lysate was purified and then the protein was labelled by Biotin/DMF. The resulting biotin‐labelled proteins were diluted 1:20 in the coupling solution before being applied to the cancer phosphor‐antibody array for conjugation. The antibody microarray was blocked for 45 minutes, and then dried and incubated with the biotin‐labelled cell lysates at room temperature for 2 hours. After the array slide was washed three times, the labelled protein was detected by incubating the array in Cy3‐Streptavidin for 20 minutes in the dark. The chips were scanned using the GenePix 4000B Array Scanner (Axon Instruments, USA), and the raw data were analysed using the GenePix Pro 6.0 (Axon Instruments, USA). The analysed results were expressed by the phosphorylated protein/unphosphorylated protein ratio.^24^

### 2.8 Cell apoptosis assay and the Hoechst staining assay

Hep3B, HepG2, and SMMC‐7721 cells were treated with B10 and sorafenib for 48 hours. For flow cytometry analysis, the cells were analysed using FACS flow cytometer (Becton Dickinson, Mountain View, CA, USA). Annexin V‐fluorescein isothiocyanate/propidium iodide (FITC/PI) double staining was performed, and the Annexin V (+) cells were counted to determine the number of apoptotic cells. The obtained data were analysed using the Modfit LT software. For Hoechst staining, cells were fixed with 4% paraformaldehyde and stained with Hoechst 33258. Images were photographed under the inverted fluorescence microscope.

### 2.9 Western blot analyses

Following treatment with B10 and sorafenib for 48 hours, Hep3B, HepG2, and SMMC‐7721 cells were collected and lysed. The insoluble protein lysates were denatured and analysed for western blot analysis with primary antibodies, followed by use of the ECL kit. The Image‐Pro Plus software (Image‐Pro Plus 5.1, Media Cybernetics, Inc., Rockville, MD, USA) was used to quantify the protein.

### 2.10 RNA interference studies

Silencing RNA oligonucleotides targeting EphrinB2 were obtained from Shanghai GenePharma Co., Ltd (Shanghai, China). The EphrinB2 siRNA was designed to target the following sequence: EphrinB2, sense: 5′‐CTGCTGGATCAACCAGGAATA AAGA‐3′; antisense: 5′‐TCCTGAAGCAATCCCTGCAAATA‐3′; GAPDH, sense: 5′‐GCACCGTCAAGGCTGAGAAC‐3′, antisense: 5′‐TGGTGAAGACGCCAGTGG A‐3′. Hep3B cells were seeded to obtain a final confluency of 50%‐60%. For in vitro knockdown experiments, siRNA (in DMEM without antibiotics and FBS) was delivered at a final concentration of 80 nmol/L using the Lipofectamine 2000 reagent according to the manufacturer's protocol. After 4‐6 hours, the medium was replaced with fresh DMEM with or without different concentrations of B10 and sorafenib, and the cells were incubated for another 48 hours. Cells were harvested for the cell proliferation assay and cell apoptosis assay. The knockdown efficiency was determined by western blot 48 hours post transfection.

### 2.11 In vivo tumour suppression assay

BALB/c‐nude mice aged 4‐6 weeks were injected subcutaneously with 4 × 10^6^ Hep3B cells into the right flanks. Tumour growth was recorded by measurement of two perpendicular diameters of the tumours every other day and calculated by using the formula: volume = (length × width^2^)/2. When the tumour volume reached 80‐100 cm^3^, mice were randomly assigned to 4 groups (6 mice/group) and treated with the vehicle (0.5% CMC‐Na), B10 (20, 80 mg/kg in 0.5% CMC‐Na) or sorafenib (40 mg/kg in 0.5% CMC‐Na) every day by intragastric administration. The mouse weight and tumour volume were monitored every other day.

### 2.12 Statistical analysis

Quantitative data were expressed as means ± SEM from three separate experiments for each condition. Statistical analysis was performed using the one‐way ANOVA and further Tukey's multiple comparison test was used to analyse statistical differences between groups under different conditions. For all other data, an independent‐samples *t* test was used. A *P‐value < .05 was considered statistically significant.

## 3 RESULTS

### 3.1 Effect of B10 on VEGFR2

B10 was synthesized by using computer‐aided drug design, which was a tool for designing novel compounds for protein targets. In order to confirm whether B10 could inhibit the activity of VEGFR kinase in vitro, the phosphorylation of a peptide substrate by VEGFR kinase was evaluated in a microtitre plate format using LANCE. As shown in Figure S1a, B10 treatment attenuated VEGFR2 activity in a dose‐dependent manner and the IC50 of B10 to VEGFR2 kinase was 2.150** **μmol/L (IC50 of sorafenib to VEGFR2 kinase was 1.329 μmol/L), suggesting that B10 altered VEGFR2 kinase activity effectively.

Cell membrane chromatography (CMC) method is an effective technique to study the characteristics of drug‐membrane receptor affinity.^25^ Elution profiles of B10 and sorafenib on VEGFR2/HEK293 CMC columns are shown in Figure 1B. The retention behaviour indicated that both B10 and sorafenib could bind to VEGFR2. Furthermore, the retention time of B10 was 38.01 minutes and that of sorafenib was 11.42 minutes (Figure 1C).

### 3.2 EphrinB2 expression correlates with human liver cancer cell lines

To investigate the clinical significance of EFNB2 in liver cancer, we firstly analysed the EFNB2 gene in the TCGA database to examine the correlation of EFNB2 with normal and clinical liver tissues. EFNB2 was significantly highly expressed in liver cancer tissues than in normal tissues. As shown in Figure 1D, there was a significant difference in EFNB2 expression between normal and clinical liver tissues (*P* < .0001). Next, we conducted a study to detect EFNB2‐related protein EphrinB2 expression levels in liver cancer cells by western blotting. Results showed that EphrinB2 was highly expressed in liver cancer cell lines (Figure 1E). Taken together, these results indicate that EphrinB2 expression is closely related to liver cancer development.

We then used the CMC method to evaluate B10 and EphrinB2 receptor affinity. Elution profiles of B10 and sorafenib for EphrinB2/HEK293 CMC columns were shown in Figure 1F. B10 in the effluent could combine with the immobilized receptor EphrinB2, which was present at the stationary phase surface. Furthermore, the retention time of B10 (56.39 minutes) was longer than that of sorafenib (11.22 minutes) (Figure 1G). Therefore, B10 could bind to EphrinB2, and their combined strength is higher than that of sorafenib.

### 3.3 B10 inhibits liver cancer cell growth and colony formation

**Figure 2:**
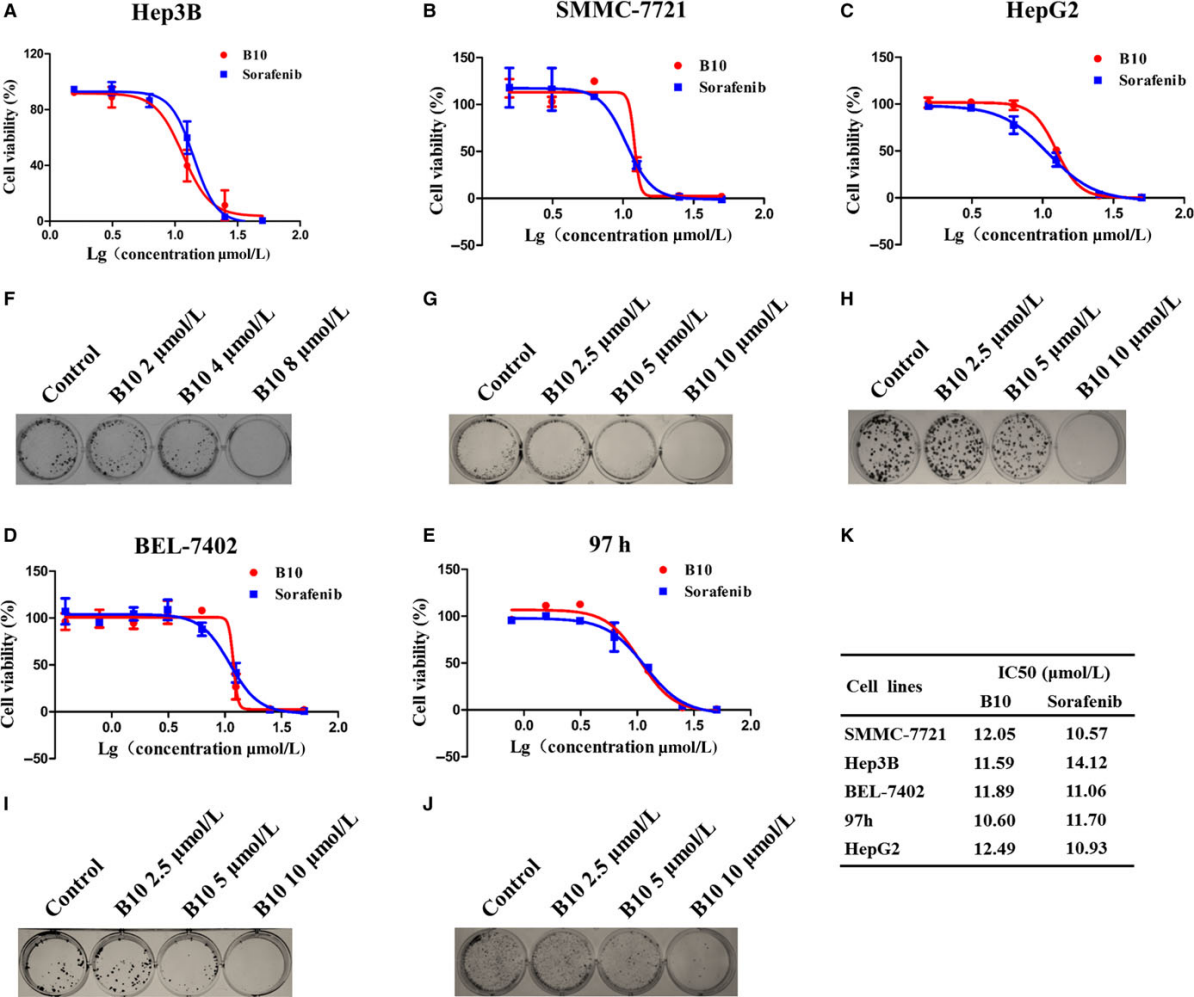
B10 suppresses liver cancer cell proliferation and colony formation. A‐J, Effect of B10 on liver cancer cell proliferation and colony formation: (A, F) Hep3B, (B, G) SMMC‐7721, (C, H) HepG2, (D, I) Bel‐7402 and (E, J) 97 h. K, The IC50 value of B10 for inhibiting liver cancer cell proliferation

Based on the high expression of EphrinB2 in liver cancer cell lines, we used MTT and colony formation assays to examine the effect of B10 on cell proliferation and colony formation in a panel of liver cancer cell lines, including Hep3B, SMMC‐7721, HepG2, Bel‐7402 and 97 h cells. As shown in Figure 2A‐E, we found that B10 significantly inhibited liver cancer cell proliferation and anchorage‐dependent growth in a dose‐dependent manner (Figure 2F‐J). In addition, IC50 values of B10 for SMMC‐7721, Hep3B, Bel‐7402, 97 h and HepG2 cells at 48 hours were 12.05, 11.59, 11.89, 10.60 and 12.49 μmol/L (Figure 2K). These findings indicate that B10 exhibits potential anti‐tumour properties in liver cancer cells.

### 3.4 Effect of B10 on EphrinB2

**Figure 3:**
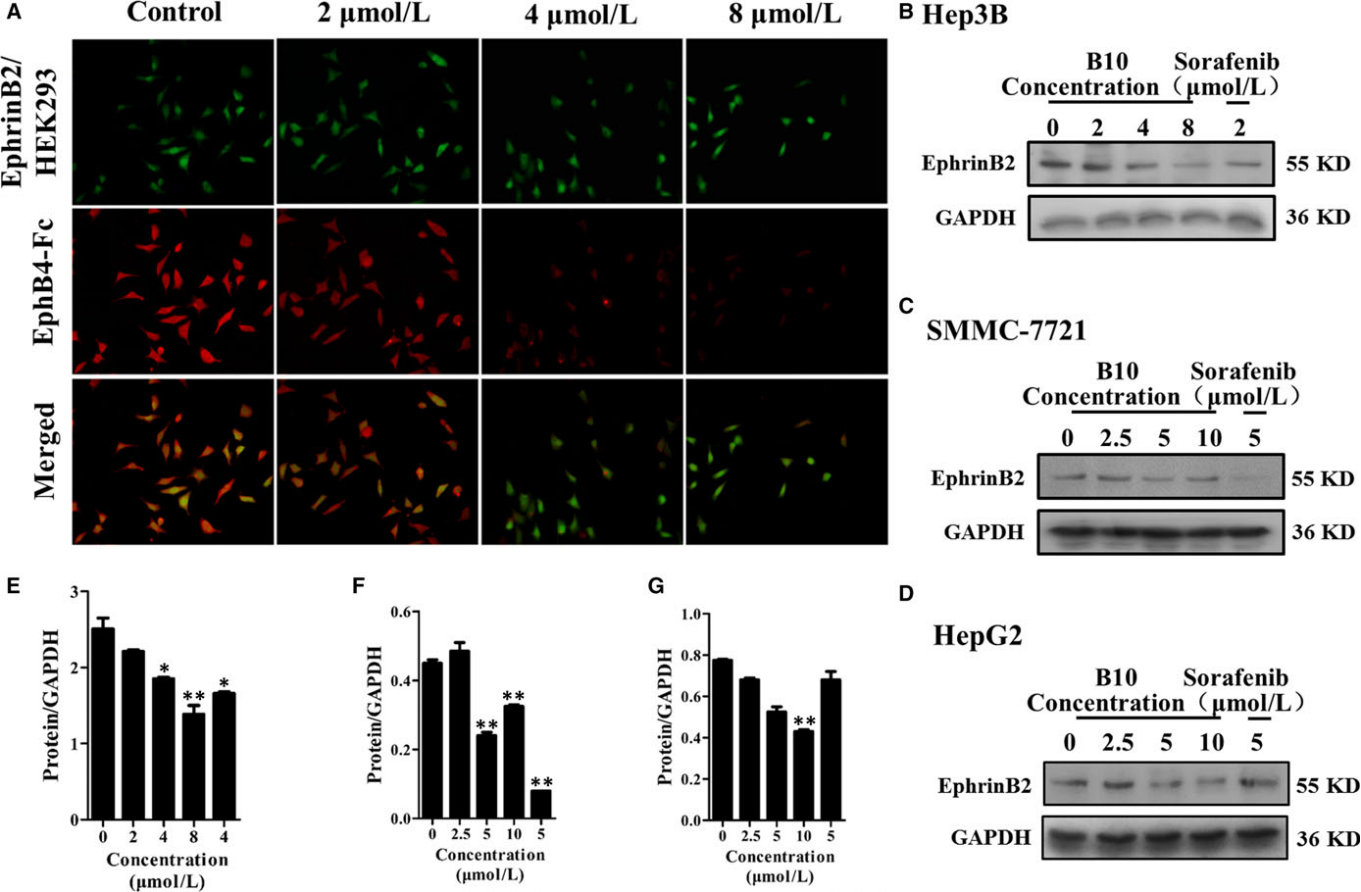
Effect of B10 on EphrinB2. A, Effect of B10 on the subcellular localization of EphB4‐Fc cultured with EphrinB2/HEK 293. B‐D, Effect of B10 on EphrinB2 expression on liver cancer cell included Hep3B, SMMC‐7721 and HepG2. E‐G, Quantification of (B‐D). Values are presented as mean ± SEM. *P <* *.05, **P <* *.01 vs the control group

Furthermore, we used the fluorescent competitive study to explore whether B10 could competitively bind to the site on EphrinB2 occupied by EphB4. Results showed that EphB4 expression was decreased (Figure 3A). This indicated that B10 could compete with EphB4 for binding to EphrinB2.

We also detected the effect of B10 on EphrinB2 expression in liver cancer cells, including Hep3B, SMMC‐7721 and HepG2 cells. As shown in Figure 3B‐D, B10 could effectively down‐regulate EphrinB2 expression in these cells.

### 3.5 Differentially expressed proteins induced by B10 and siRNA EphrinB2

**Figure 4:**
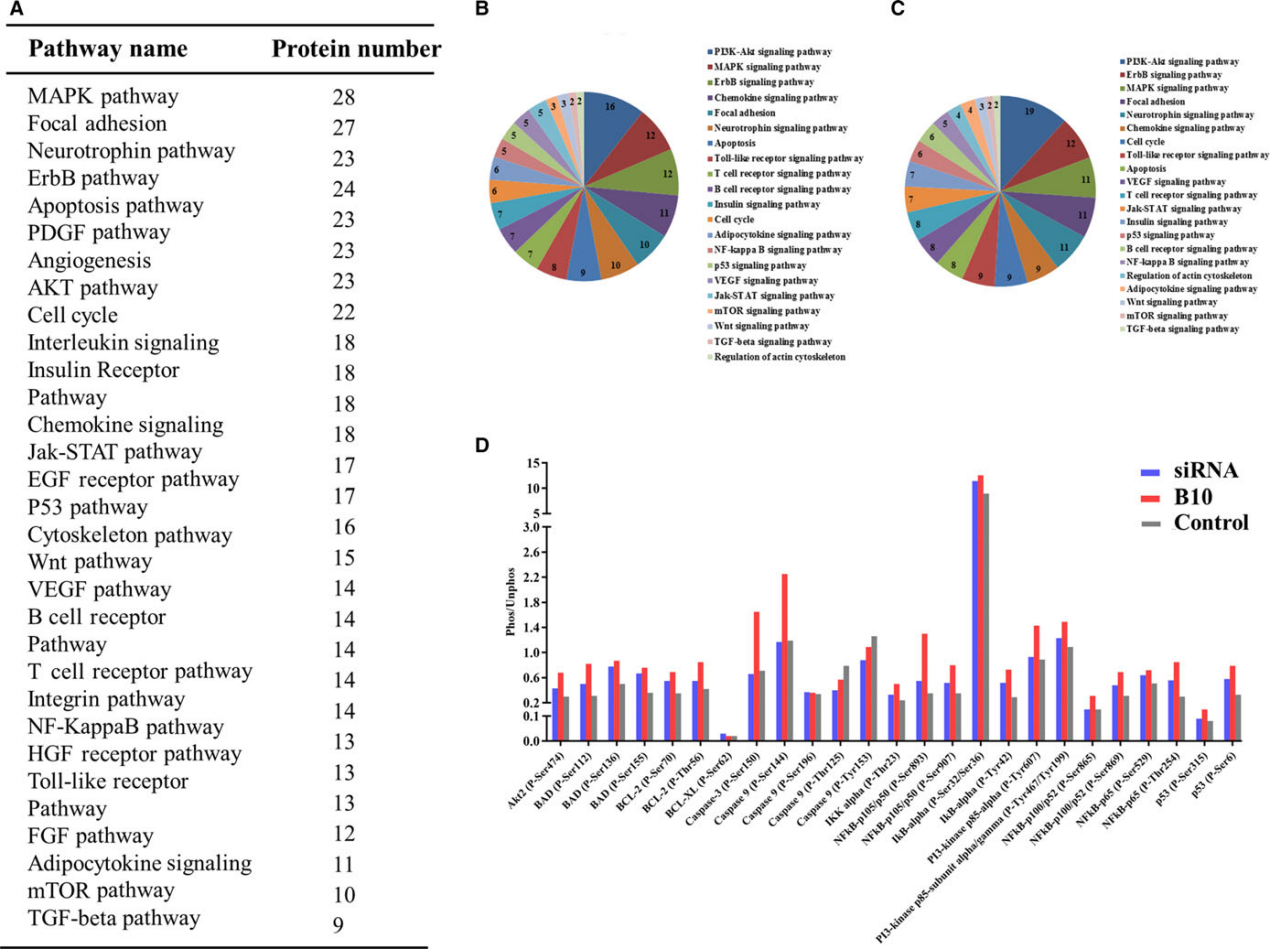
Protein microarray. A, Different pathways detected in the Protein Chip assay. Differentially activated signalling pathways associated with several cancer biological processes induced by B10 (B) and siRNA EphrinB2 (C). Proteins obtained from the array were further analysed by KEGG and pathway mapping analysis. D, Apoptosis‐related proteins induced by B10 and siRNA EphrinB2

To comprehensively determine the mechanism of B10 functioning, we performed a phosphor‐proteomics‐based study using a phospho‐antibody microarray (Full Moon BioSystems Inc.), which provides a high‐throughput platform for efficient protein phosphorylation status profiling, with detection and analysis of phosphorylation events at specific sites to identity pathways that are regulated by B10 and EphrinB2 knockdown (Figure 4A). In total, 42 differentially phosphorylated proteins were identified between control and B10 treated Hep3B cells when the fold‐change was ≥2; 52 differentially phosphorylated proteins were identified between control and siRNA of EphrinB2 and Hep3B cells when the fold‐change was ≥1.5. Proteins obtained from the array were further analysed by KEGG and pathway mapping analysis. Results indicated that these proteins were associated with several cancer biological processes such as cell growth, cell apoptosis and cell migration (Figure 4B,C). Interestingly, both B10 treatment and siRNA EphrinB2 induced up‐regulation of pro‐apoptosis protein phosphorylation (Figure 4D). Therefore, EphrinB2 might be an anti‐apoptotic protein, and B10 could induce cell apoptosis.

### 3.6 B10‐induced liver cell apoptosis

**Figure 5:**
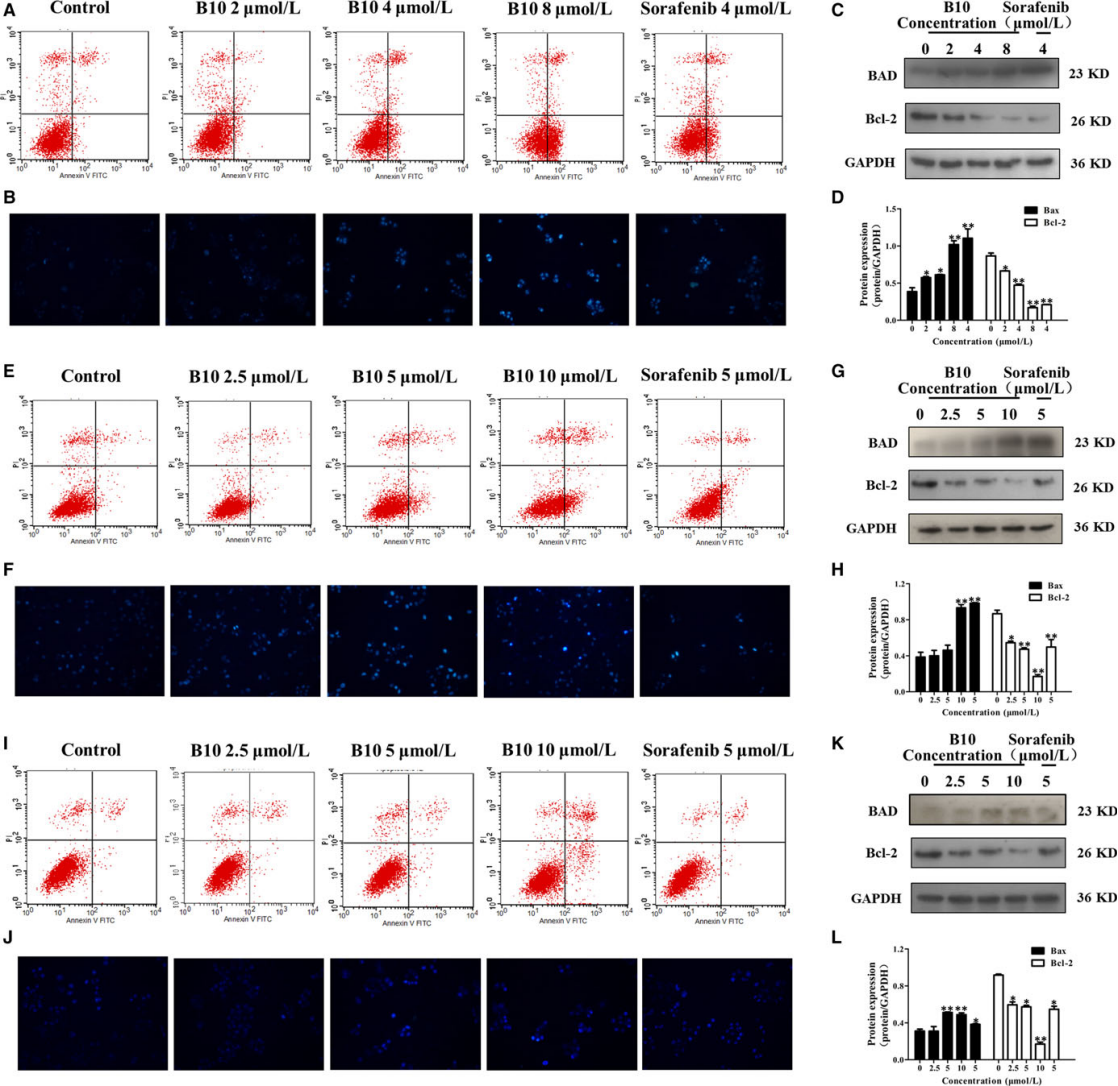
B10 induces liver cancer cell apoptosis. A, E, I, The flow cytometry profile represents Annexin V‐FITC and PI staining: (A) Hep3B cells, (E) SMMC‐7721 cells, and (I) HepG2 cells. B, F, J, Apoptosis level after B10 treatment assessed by Hoechst 33258 staining: (B) Hep3B cells, (F) SMMC‐7721 cells, and (J) HepG2 cells. C, G, K, Western blot analysis of Bad and Bcl‐2 expression in Hep3B, SMMC‐7721, and HepG2 cells. D, H, L, Results were quantified by densitometric analysis of bands from C, G and K. Values are presented as mean ± SEM. *P <* *.05, **P <* *.01 vs the control group

To confirm the phosphor‐antibody array, we used a combination of flow cytometry analysis and Hoechst 33258 staining to monitor human liver cell apoptosis both with and without treatment of B10 and sorafenib. As shown in Figure 5A,E and I, FACS results showed that in Hep3B, SMMC‐7721, and HepG2 cells treated with B10, cell apoptosis increased in a dose‐dependent manner. Following treatment with 0, 2, 4 and 8 μmol/L of B10 for 48 hours, the percentage of apoptotic cells was 6.78%, 5.38%, 15.24% and 40.4%, respectively, among Hep3B cells. The percentage of apoptotic cells was 4.46%, 4.6%, 9.36% and 21.1% among SMMC‐7721 cells, following treatment with 0, 2.5, 5 and 10 μmol/L of B10 for 48 hours. The percentage of apoptotic cells was 2.62%, 3.68%, 3.7% and 14.98% among HepG2 cells, following treatment with 0, 2.5, 5 and 10 μmol/L of B10 for 48 hours respectively.

Hoechst 33258 staining, commonly used to stain the genomic DNA of Hep3B, SMMC‐7721 and HepG2 cells, showed that B10 treatment induced apoptotic events characteristic of chromatin condensation. Microscopic observations in Figure 5B,F and J demonstrated a typical morphology of apoptotic nuclei stained with Hoechst stain, in which chromatin was condensed and aggregated at the nuclear membrane by a bright fluorescence at the periphery. Our data suggest that B10 could induce apoptosis in Hep3B, SMMC‐7721 and HepG2 cells.

We examined Bad and Bcl‐2 expression among the cell apoptosis molecules in the Bcl‐2 family. Results showed that Bad protein expression gradually increased following B10 treatment. Meanwhile, the Bcl‐2 expression was significantly down‐regulated in B10 treated Hep3B, SMMC‐7721, and HepG2 cells (Figure 5C,D,G,H,K and L). These results are consistent with those of phospho‐antibody array analysis.

### 3.7 EphrinB2 modulates cell apoptosis in liver cancer cells

**Figure 6:**
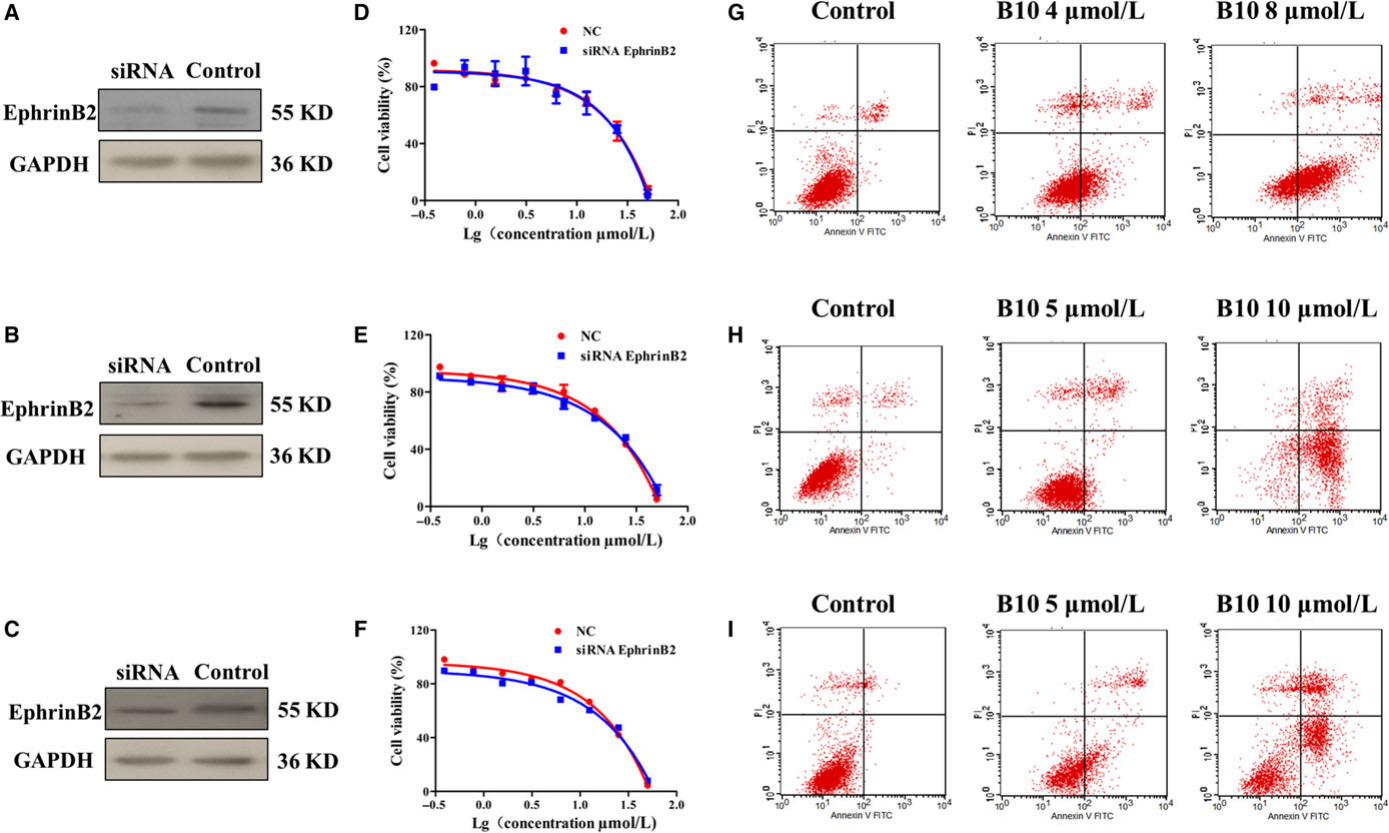
Effect of B10 on the apoptosis of cells transfected with siRNA EphrinB2 and wild‐type cells in vitro. A‐C, Western blot analysis of EphrinB2 in Hep3B, SMMC‐7721 and HepG2 cells subjected to EphrinB2 knockdown. D‐I, Hep3B, SMMC‐7721 and HepG2 cells were transfected with siRNA against EphrinB2 followed by B10 treatment. Cell proliferation and apoptosis was assessed by (D‐F) MTT assay and (G‐I) flow cytometry analysis, respectively. Values are presented as mean ± SEM. *P <* *.05, **P <* *.01 vs the control group

We further established liver cancer cell lines in which EphrinB2 was inhibited via siRNA expression. These cell lines were used to determine whether EphrinB2 could affect cell apoptosis. As shown in Figure 6A‐C, the protein level of EphrinB2 was down‐regulated in EphrinB2‐silencing cells. Cell proliferation was determined at 48 hours after seeding was done using an MTT assay. As shown in Figure 6D‐F, Hep3B, SMMC7721 and HepG2 cell proliferation remained unchanged upon a decrease in EphrinB2 level, after silencing of endogenous EphrinB2. However, knockdown of EphrinB2 by siRNA significantly enhanced the apoptotic induction of B10 (Figure 6G‐I). Following treatment with 0, 4, and 8 μmol/L of B10 for 48 hours in Hep3B cells knock‐down EphrinB2, the percentage of apoptotic cells was 4.2%, 30.06% and 65.5%, respectively. The percentage of apoptotic cells was 2.54%, 25.89% and 42.64% among SMMC‐7721 cells knock‐down EphrinB2, following treatment with 0, 5 and 10 μmol/L of B10 for 48 hours. The percentage of apoptotic cells was 3.52%, 12.82% and 50.36% among HepG2 cells knock‐down EphrinB2, following treatment with 0, 5 and 10 μmol/L of B10 for 48 hours respectively. This indicated that EphrinB2 was a key factor in apoptosis induction by B10.

### 3.8 Effect of B10 on molecular of EphrinB2 signalling pathway

**Figure 7:**
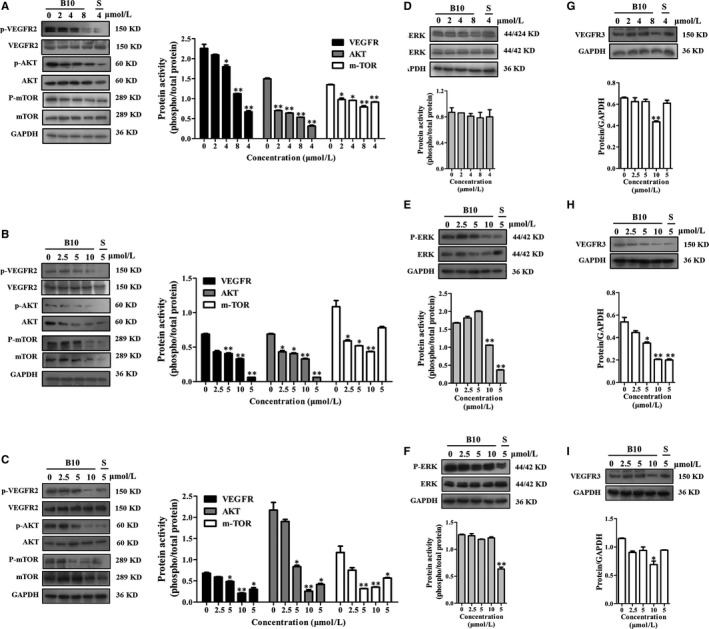
Effect of B10 on expression of the EphrinB2 signalling pathway. A, B, C, Western blot analysis of VEGFR2, AKT and mTOR protein expression in (A) Hep3B cells, (B) SMMC‐7721 cells and (C) HepG2 cells with quantification data. D, E, F, Western blot analysis of ERK1/2 protein expression in (D) Hep3B cells, (E) SMMC‐7721 cells and (F) HepG2 cells with quantification data. G, H, I, Western blot analysis of VEGFR3 protein expression in (G) Hep3B cells, (H) SMMC‐7721 cells and (i) HepG2 cells with quantification data. Values are presented as mean ± SEM. *P <* *.05, **P <* *.01 vs the control group

EphrinB2 signalling has been reported to regulate the internalization of VEGFR2 and VEGFR3 activity. VEGFR2 downstream signalling pathways include both PI3K/AKT/mTOR and MAPK signalling cascades, which play an important role in proliferation, angiogenesis and metastasis of tumour cells. To identify the effect of B10 on EphrinB2 and its downstream signalling pathways that might contribute to growth inhibition further, we examined the phosphorylation of several key regulators by western blotting. The results showed that treatment with B10 significantly decreased the phosphorylation of VEGFR2 and its downstream signalling members (AKT, mTOR and ERK1/2) in Hep3B, SMMC‐7721 and HepG2 cells (Figure 7A‐F). Meanwhile, we found that B10 inhibited the expression of VEGFR3 (Figure 7G‐I). These results suggested that the inhibition of EphrinB2 simultaneously interfered with the functioning of VEGFR2 and VEGFR3.

### 3.9 B10 has a potent antitumour effect in vivo

**Figure 8:**
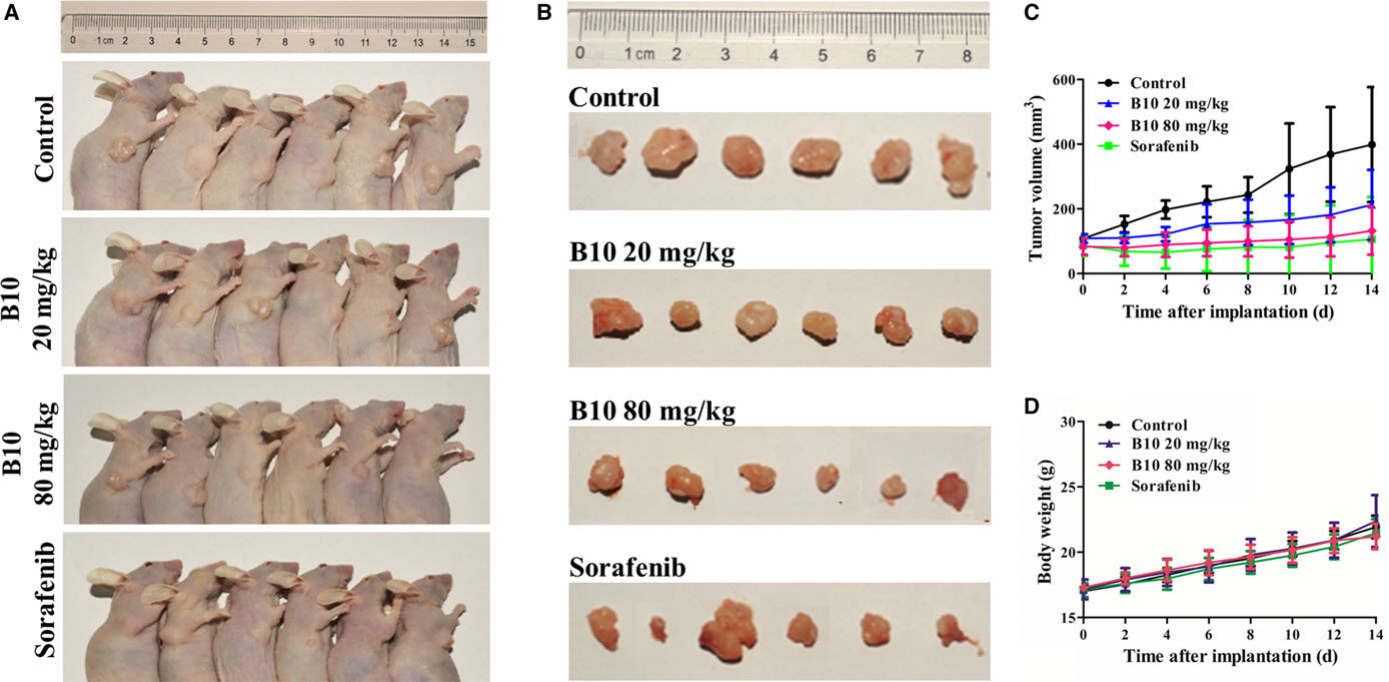
B10 inhibits tumour growth of Hep3B cells in a xenograft mouse model. A, Xenograft model in nude mice. Six different sets of nude mice were injected subcutaneously. Representative pictures of mice in each group are shown. B, Tumour tissues from representative mice in each group. C, Tumour growth over time was measured in each group at the indicated time point for various treatments. D, Animal weight changes over time were determined for each group every other day. Data are expressed as the mean ± SD (n = 6 mice per group)

To test the efficacy of B10 on tumour growth in vivo, a xenograft tumour model was established in nude mice. The anticancer effects of B10 against Hep3B transplanted tumours are shown in Figure 8A. The results indicate that Hep3B cell‐derived xenograft tumours progressively grew in the control group, whereas tumours grew slowly in the mice treated with B10 and sorafenib. Tumour volume was significantly lower in B10‐treated mice and sorafenib‐treated mice than in control mice (Figure 8B). On day 15, all of the mice were killed, and tumours were excised and weighed. These results showed that the average tumour weights with the vehicles B10 (20 mg/kg), B10 (80 mg/kg) and sorafenib (40 mg/kg) treatment groups were 0.34 ± 0.15, 0.20 ± 0.06, 0.15 ± 0.12 and 0.17 ± 0.17 g respectively (Figure 8C). It was found that 20 and 80 mg/kg B10 inhibited Hep3B xenograft tumour growth by 42.62% and 56.07% and that sorafenib (40 mg/kg) had an inhibitory effect of 50.37%. However, there were no significant changes in the body weight of mice during these experiments between these four groups. The final average body weights were 21.93 ± 0.86, 22.38 ± 1.98, 21.2 ± 0.96 and 21.47 ± 1.07 g in the control, B10 (20 mg/kg), B10 (40 mg/kg) and sorafenib groups respectively (Figure 8D). There were no statistically significant differences in average body weights in different groups. Collectively, these results validate that B10 might be a safe and effective agent for liver cancer therapy, at least in xenograft tumour models.

## 4 DISCUSSION

Hepatocellular carcinoma is the third leading cause of deaths occurring because of cancer worldwide.^26^ Currently, most HCC patients cannot be diagnosed in the early stage, and therefore, they lose the best opportunities for treatment. Despite tremendous advances in early diagnosis and surgery, HCC incidence continues to increase worldwide. Therefore, novel preventive strategies and therapeutic approaches for HCC urgently need to be discovered and developed.

B10 was synthesized to target VEGFR2 by computer‐aided drug design, a tool for designing novel compounds for protein targets. We found that B10 could inhibit the VEGFR2 kinase activity in vitro by evaluating the phosphorylation of a peptide substrate by VEGFR2 kinase in a microtitre plate format using LANCE. In addition, the elution profiles of B10 and sorafenib on the HEK293/VEGFR2 CMC column indicated that B10 and sorafenib could bind to VEGFR2. It is well‐known that VEGFR2 plays a vital role in tumour angiogenesis and metastasis.^27^ EphrinB2, which is expressed in cancer cells, was proven to be involved in VEGF/VEGFR mediated angiogenesis.^28^ We address this issue by examining the correlation between the expression of EphrinB2 and the progression of liver cancer. Here, we examined the expression of EFNB2 in liver cell carcinoma specimens by using RNA‐Seq data from 292 paired tumour and adjacent normal samples obtained from The Cancer Genome Atlas. The results revealed that EFNB2 overexpression was higher in cancerous liver tissue than normal tissue. It indicated that EFNB2 was overexpressed in human liver cancer, and the overexpression was correlated with tumour progression and poor patient outcome. Additionally, western blot results showed that EphrinB2 was predominantly expressed in a spectrum of human liver cancer cell lines, including Hep3B, SMMC‐7721, HepG2, Bel‐7402 and 97 h cells. A high expression of EphrinB2 was associated with poor prognosis and tumour progression. Therefore, EphrinB2 may serve as a therapeutic target for treating liver cancer cells. Because sorafenib is the only targeted drug approved for treating advanced HCC,^29^ we used sorafenib as a positive control in our study.

In this study, MTT and colony formation analysis were performed to detect the effect of B10 on the regulation of liver cancer cell proliferation. We found that B10 significantly reduced cancer cell growth and colony formation in a panel of cells, including Hep3B, SMMC‐7721, HepG2, Bel‐7402 and 97 h cells, which showed high EphrinB2 expression. Moreover, we demonstrated that B10 could bind to EphrinB2 by the CMC assay. Fluorescent competitive binding assays further validated that B10 might have direct competition for a single common binding site on EphrinB2. Additionally, our results indicated that B10 down‐regulated the EphrinB2, Hep3B, SMMC‐7721 and HepG2 cells at the protein level. These results suggest that B10 might target EphrinB2.

To gain insights into the mechanisms by which B10 bound to HCC is inhibited, a phosphor‐antibody array was used to screen the potential targets and signalling pathways. The major findings from array analysis were that differentially phosphorylated proteins associated with apoptosis and growth were obtained after B10 treatment. Such proteins are associated with the PI3K‐Akt, MAPK, VEGF and mTOR signalling pathways. Importantly, we found that the phosphorylation of pro‐apoptotic proteins were up‐regulated after B10 treatment. Interestingly, array analysis showed that siRNA knockdown of EphrinB2 could lead to an increase in apoptosis. It indicated that EphrinB2 might serve as an anti‐apoptotic protein. Previous research has consistently offered the information that EprinB2 might induce the apoptosis of vascular endothelial cells during pathological neovascularization.^30^, ^31^ We further confirmed the array data by flow cytometric and Hoechst 33258 staining assays. It was shown that B10 induced apoptotic events that were characteristic of the significantly increased apoptotic fraction and chromatin condensation on Hep3B, SMMC‐7721 and HepG2 cells. It was determined that a further insight into the mechanism by which B10 affects the Bad and Bcl‐2 protein expression was required. The results showed that B10 up‐regulated Bad expression, which was correlated with the down‐regulation of Bcl‐2 expression. Meanwhile, combining B10 with siRNA enhanced apoptotic induction. Indeed, an antiapoptotic role of EphrinB2 is consistent with the increased apoptosis in Hep3B, SMMC‐7721, and HepG2 cells observed by flow cytometric analysis. This also suggests that EphrinB2 might be a target forB10. EphrinB2 promotes VEGFR endocytosis in endothelial cells, thereby enhancing VEGF‐mediated angiogenesis,^32^ which is essential in normal and pathological situations.^33^ It has been implicated in mediating angiogenesis signalling in human cancer cells by promoting the internalization of VEGFR2 and VEGFR3, which in turn are linked to downstream effectors involved in PI3K/AKT/mTOR and ERK/MAPK pathways.^34^, ^35^ The proteins selected by phosphor‐antibody array were confirmed by western blot. As expected, our results showed that B10 inhibited the phosphorylation of VEGFR2 and the expression of VEGFR3. Meanwhile, B10 decreased the activation of VEGFR2 downstream signal molecules, including the activation of AKT, mTOR, and ERK1/2 in Hep3B, SMMC‐7721, and HepG2 cells. Most importantly, B10 caused an effective inhibition of tumour cell growth in the xenografts in athymic mice.

In summary, this study reports that EphrinB2, which is overexpressed in liver cancer, could serve as an apoptosis promoter, and hence, it plays a functional role in liver cancer. B10 has an inhibitory effect on HCC cells, whose underlying mechanism is via the targeting of the EphrinB2 signalling pathway and apoptosis induction. It represents a promising anticancer agent for conducting further clinical trials, for the use of B10 in liver cancer treatment.

## CONFLICT OF INTEREST

The authors declare that they have no conflicts of interest to disclose.

## AUTHOR CONTRIBUTIONS

BLD, XPS and NM performed the main experiments, summarized the results. WNM and TFY assisted in performing the experiments. YMZ assisted in interpretation the data. JZ supplied the compound. BLD wrote the manuscript. BLD and LCH provided the concept, funding, supervision and assisted in writing the manuscript. All authors read and approved the final manuscript.
