# Supplementary material for: HMQ‐T‐B10 induces human liver cell apoptosis by competitively targeting EphrinB2 and regulating its pathway
Source: J Cell Mol Med. 2018 Sep 14;22(11):5231–43. doi: 10.1111/jcmm.13729 (PMC6201340; doi:10.1111/jcmm.13729)
Supplement: Supplementary file 2 [file JCMM-22-5231-s002.docx]

**Supplement figure legends**

**Supplementary Fig. S1. Effect of B10 on VEGFR2** **kinase activity**.
